# Evaluation of an Early Childhood Parenting Programme in Rural Bangladesh

**Published:** 2007-03

**Authors:** Frances E. Aboud

**Affiliations:** ICDDR,B, GPO Box 128, Dhaka 1000, Bangladesh and Department of Psychology, McGill University, Montreal, Canada H3A 1B1

**Keywords:** Parenting, HOME, Child development, Cognitive development, Child health, Stimulation, Evaluation studies, Bangladesh

## Abstract

To promote physical and mental development of children, parenting education programmes in developing countries focus on specific practices such as age-appropriate responsive stimulation and feeding. A programme delivered to groups of poor mothers of children, aged less than three years, in rural Bangladesh was evaluated using an intervention-control post-test design. Mothers (n=170) who had attended a year of educational sessions and their children were compared with those (n=159) from neighbouring villages who did not have access to such a programme. After covariates were controlled, the parenting mothers obtained higher scores on a test of child-rearing knowledge and on the Home Observation for Measurement of the Environment (HOME) inventory of stimulation. The parenting mothers did not communicate differently with their children while doing a picture-talking task, and children did not show benefits in nutritional status or language comprehension. Parenting sessions offered by peer educators were informative and participatory, yet they need to include more practice, problem-solving, and peer-support if information is to be translated into behaviour.

## INTRODUCTION

The importance of care and stimulation of children, aged less than three years, has become especially critical as more children survive and their quality of life becomes a concern. Although still inconclusive, it appears that rapid growth in the brain during these early years may dissipate if unused (1). Various programmes are being implemented around the world with the objective of fostering conditions that optimize child growth and development (2). The most common programme in developing countries is a parenting education programme addressed to mothers with or without a child component (3). Its aim is to foster more mother-child interaction for the purposes of stimulation and nutrition. Although many parenting programmes are implemented by organizations in developing countries, few are ever evaluated (4), especially in South Asia (5, 6). It is important to evaluate their effectiveness so that organizations which provide parent education to groups, similar to the one described here, can create an effective model. The present study contributes to this ongoing effort by evaluating the effectiveness of a parenting programme developed in Bangladesh for poor rural mothers. Using a post-test only intervention-control design, we evaluated outcomes, such as mothers' home stimulation and child vocabulary, along with the educational process used in parenting sessions.

The rationale for parenting programmes is two-fold. The first is that parents need to be involved when targeting child development because their sensitive responsiveness is crucial to secure attachment and its multiple consequences (7). The second is that when children are at risk for poor language and cognitive development (8), opportunities for stimulation and learning must be created at home if children do not attend preschool. In rural Bangladesh, the need for early intervention is pressing. Some 48% of children aged less than five years are moderately or severely malnourished (9). Malnutrition is strongly associated with lower cognitive and language development in Bangladesh as elsewhere (10, 11). However, stimulation with or without food supplements benefits mental development more in the long term than food by itself (12). Yet, illiterate parents are often uninformed about the need for stimulating experiences to enhance development (13). Parenting programmes can fill this gap by providing new information and demonstrating new practices for mothers of young children.

Little is known about parenting practices in rural Bangladeshi families. A recent survey found that almost half the rural mothers had no education and that most were unaware of the importance of fostering curiosity and self-confidence in a child (14). The most commonly-mentioned maternal behaviours for promoting mental development in children aged less than three years were giving nutritious food (26%) and teaching a child to talk (21%); providing opportunities for play and conversation were rarely mentioned. Home-observations and maternal recall of daily activities of children aged 3–5 years supported survey findings in that children spent many hours by themselves with few materials (15). Despite this, parents want their children to excel at school and enroll over 80% in primary schools. Consequently, Bangladeshi parenting programmes focus on informing mothers about a home environment that promotes physical and mental development.

The parenting programme evaluated here was part of the offerings of a non-profit organization operating in poor areas of rural Bangladesh. The parenting programme for mothers of children aged less than three years is conducted in hundreds of villages. It involves 90-minute weekly education sessions offered by trained women, known as facilitators, to groups of 20 or so mothers. The topics include: common diseases and oral rehydration solutions, hygiene, sanitation, breastfeeding, weaning foods, micronutrient deficiencies, stages of cognitive and language development, how parents can help children learn, how to encourage language development, positive discipline, gender equality, and child rights (16). For example, the nutrition topic includes foods to feed and how to make food appealing to a child; the stimulation topic includes how to make home-made toys and talk to a child while you work; and the hygiene topic covers latrine use and bathing. The programme was developed by Bangladeshi experts in health and early childhood to be culturally appropriate and feasible for a poor rural population, for example by itemizing foods in season and learning materials available in rural villages. At the time, it had been functioning for several years and revised once to add more topics. The facilitators had some secondary education; to deliver the programme, they additionally received 17 days of basic training with a manual of 40 topics, four days a month of supervision, and monthly refresher courses.

Although the content of a successful parenting programme has not been firmly defined, studies have identified critical parenting practices and ways of measuring them (17). These include provision of responsive stimulation, language, hygiene, and a varied diet—all included in this programme. Although parenting practices were the focus of the educational format, the explicit goal was to improve children's health, growth, and development. Consequently, outcomes relating to maternal knowledge and practices, and child language and nutritional status were assessed.

Less agreement is found on the methods needed to change maternal behaviours. According to some reviews, programmes are more likely to change behaviours if they include some information, opportunities to observe role models and to practise the skills, participatory problem-solving, focused goals, use of peer educators, and a minimum of 14 hours of contact (18, 19). The behaviour-change strategy most commonly used in parenting programmes entails the provision of information and advice [although some add demonstrations and community supports (20)]. The one evaluated here was not guided by a behaviour-change theory, but as with others it was consistent with the ecological models that combine psychological theories of behaviour with public-health breadth (21). The assumption is that information and advice will be translated into behaviour. Group sessions are expected to enhance social motivation to participate. So, although no standard measure exists to evaluate programmes, we evaluated features of the sessions.

As independent evaluators of the programme, our objectives were to: (a) examine the impact of the parenting programme on parenting knowledge and practices of mothers, especially practices that concern psychosocial stimulation; (b) examine the impact of the parenting programme on children's language development and nutritional status; (c) determine whether mothers with more or less education benefited more than others from the programme; and (d) assess the quality of the programme in terms of the active and participatory nature of the sessions themselves and the opportunities to observe role models and to practise the skills. These results could then be used for informing the parenting programmes that use this model on how well they reach their stated goals of improving practices of mothers and child outcomes.

## MATERIALS AND METHODS

### Study design

The study used a post-test only intervention-control design. Mothers who attended parenting sessions in the previous year and their children where compared with controls from nearby villages where parenting sessions were not available. The parenting programme had finished two months prior to data collection. The Research Review Committee and the Ethics Review Committee of ICDDR,B approved the protocol. Plan International Bangladesh provided funds to conduct the study.

### Study population, recruitment, and sample

Three rural districts were chosen where Plan International Bangladesh had parenting sessions in sufficient numbers. Sample sizes were estimated according to the expected mean language scores of 10 out of 20 with a standard deviation of 1.5. Setting alpha=0.05 and power=0.90, an n of 150 for parenting and control groups provided enough power to detect a mean difference of half a standard deviation.

Mothers and children were recruited from 22 parenting and 22 control villages in the following manner. First, villages where parenting sessions were conducted during the previous year were randomly selected; the mothers on the list were then visited to determine if their children were aged 2.5–4.0 years (30–48 months). This would mean that the mother had attended the programme, while her child was aged 2 or 3 years. If the child fit the age-eligibility criterion, the mother was invited to participate. Research assistants could select at most eight mothers and children from the same parenting group. Control villages were ones where Plan International Bangladesh had activities but no parenting groups. In control villages, the research assistants started from three different points in the village, asking families if they had a child within the age range. If they did, they were recruited. Sociodemographic similarities between the groups were statistically examined. Consent was obtained from mothers before the interview. All parenting mothers agreed to participate and approximately 95% of control mothers. The sample included 170 parenting mothers and their children (99 boys, 71 girls) and 159 controls (73 boys, 86 girls) for a total of 329.

### Measurement of mother variables

All measures were translated into Bangla and were back-translated; discrepancies were resolved with the help of bilingual Bangladeshis familiar with similar measures.

#### Family sociodemographic status

The mothers reported on the household members, their age, sex, educational attainment, and occupation. The economic status was assessed with Yes-No questions about the ownership of 11 assets commonly included in the Bangladesh Health and Demographic Survey (9), such as table, bed, radio, and electricity, ownership of a homestead and of land for production. The sum of all assets recorded as Yes had an alpha of 0.79 and correlated highly with income, owning land for production, education of mother, and education of father: r's=0.51, 0.40, 0.56, and 0.51 respectively, n=329, p<0.0001. Some 96% owned their homestead; so, this indicator showed little variability. Thus, the total number of assets was used as the economic status indicator of the family.

#### Knowledge of mothers about good practices

Knowledge of the mothers about good practices for child development was assessed with 17 open-ended questions scored from 0 to 3; after each response, the mother was prompted with ‘What else?’ until she gave 3 or could offer no more. They were taken from topics and information found in the Parenting Manual. Any good answer was given a point for a maximum of 3. For example, the following answers to “what parents can say to help their child learn” each received a point: ask questions, teach numbers, and teach words. These answers to “how play benefits a child” received a point: learns to sing, learns to get along with others, and learns colours. Keeps a child quiet did not earn a point. The responses were factor analyzed, and alpha coefficients were calculated to determine which items fit a unitary construct of knowledge. Four items were dropped, and 13 retained with an alpha of 0.66. Consequently, the range of scores was 0 to 39.

#### Parenting evaluation

The number of days the mother had attended parenting sessions was recorded and verified with the attendance lists. Mothers who attended were asked their opinion on what new they had learned (tallied but not analyzed), and their evaluation of the parenting experience as very good (3), good (2), more or less good (1), or not good (0).

#### Home Observation for Measurement of the Environment

The Home Observation for Measurement of the Environment (HOME) (22) is commonly used for measuring the amount and quality of stimulation and support provided to a child in the family setting (23, 10). A modified version of the infant-toddler inventory has 45 items which are to be scored based on observation wherever possible and otherwise on answers of mothers to questions. Factor analyses did not yield the usual six factors. However, 14 items from the learning materials and involvement subscales loaded on the first factor and together had an alpha coefficient of 0.79. They were, therefore, summed to create a subscale called stimulation which was analyzed along with the total HOME score.

#### Mother-child interaction during picture task

To evaluate the role of the mother as a mediator of cognitive development of her child, we developed a task where the mother interacted verbally with her child (24, 25). The picture task required the mother and child to talk as they normally would about two provided coloured pictures of scenes from rural Bangladesh. The pictures were on two sides of a laminated sheet. One was a rural village scene, and the second was a series of eight paintings of men and women engaged in productive activities, such as driving a rickshaw, selling at the market, and embroidering. The task was allotted five minutes. Two assistants sitting in different positions observed the interaction and tallied each mother and child utterance according to specific pre-arranged codes each time the corresponding utterance occurred. The mother codes were piloted to ensure completeness. The codes fit four levels to reflect increasingly engaging verbal stimulation as follows: Level 0—Negative evaluation, off-task/disengaged; Level 1—Command, point/name an object; Level 2—Question child, answer child, expand on detail beyond naming; and Level 3—Expand on child's behaviour, encourage child to talk or ask child to expand, positive evaluation. Child codes were included for completeness but not used for evaluating the programme because they depended too much on the mother's input. The child codes were: off-task, point, repeat mother's words, answer, name, ask, and describe detail. The mothers' speech was coded reliably: the correlations between two assistants' codes ranged from 0.55 to 0.90 with a mean of 0.79.

### Measurement of child outcomes

#### *Receptive vocabulary (WPPSI-III, 2002)* (26)

This subtest assesses children's comprehension of words. Thirty-eight words are spoken aloud, and the child is required to point to one of four pictures depicting the word. Thirteen words were substituted for the originals to maintain the expected level of difficulty within the Bangladeshi context. Scores standardized for age and ranging from 0 to 19 were used in analyses. Inter-tester reliabilities comparing scores on two different days was r(28)=0.60, p=0.0007. This is reasonable given that it reflects two testers and a test-retest difference.

#### Nutritional and health status

Children were weighed on a Uniscale to one decimal, and heights were taken. These were converted to weight-for-age, height-for-age (stunting), and weight-for-height (wasting) z-scores using the current guidelines of the Centers for Disease Control and Prevention, USA. Weight-for-height was used as a dependent variable as it would more easily increase over the course of a year as a result of the parenting sessions. Age was determined from the immunization card if possible, from a birth registration card, or from parental report with the help of a Bangla calendar and notable events. Mothers reported on preventive health behaviours relating to the child. A sum of the following five practices constituted the preventive practice score: measles immunization (a good indicator of full immunization), vitamin A drops, iodized salt, safe water, and child's latrine use. A measure of 10 questions for screening disabilities (27) provided scores from 0 to 10 to indicate the number of potential motor, sensory, speech and learning disabilities.

### Measurement of quality of parenting sessions

Observations were made of 10 current parenting sessions. These were not the sessions the participating mothers had attended; so, their quality could not be linked to previously-described mother and child outcomes. However, they were not expected to have changed much. The communicated information was not rated through observation, as this could be found in the manual. Rather, practices, problems, and participation were recorded. For example, we recorded every time a positive parenting behaviour was mentioned, along with points for whether it was elaborated, evaluated, demonstrated, and supported with materials. To assess participation, we also recorded how often mothers and facilitators raised a question and answered it, and how often they raised a problem in implementing the advice and solved it. Finally, three overall judgments were made by research assistants about the session: the talk of the facilitator was encouraging (no, yes), the session was participatory (no, yes), and the information about causes and consequences of the required behaviour took up too much time (60–100% of the session), too little (under 40%) or just right (40–60%) (18). The presence of observers can potentially influence the behaviour of facilitators and mothers. Our observers remained unobtrusive by dressing down and sitting outside the circle; however, they would likely elicit whatever facilitators thought was their best performance, not necessarily what we were recording.

### Procedure

Nine research assistants, with university degrees, received a five-day training to conduct the testing. The principal investigator and two Bangladeshi research colleagues conducted the training. At this time, inter-observer reliabilities were obtained for the vocabulary test. The trainers also observed the assistants during their first few days of data collection and on at least one other occasion during the six-week conduct of the study.

### Method of analysis

Preliminary tests were conducted to check whether the parenting and control groups differed on variables relating to demographic and socioeconomic status. The major analyses examined differences between parenting and control groups on five sets of dependent variables: mother's knowledge, the HOME inventory, mother-child talk, child vocabulary, and weight-for-height. The design was a 2 (group) × 2 (sex) analysis of covariance (ANCOVA) covarying potential confounds, namely assets, education of mother, age of child, and height-for-age. Analysis of mother-child talk additionally included, as a repeated measure, the four levels that constitute a continuum. Group × Sex interactions would indicate whether boys benefited more than girls. Means rather than adjusted means are presented as the two are almost identical. Additional analyses examined whether parenting sessions benefited one socioeconomic status group over the other. Secondary analyses were conducted on the parenting data alone to examine the quality of the programme.

## RESULTS

### Description of sample

T-test comparisons of the parenting and control groups showed no differences between the two groups on any of the potentially confounding socioeconomic status or health variables (Table 1). Almost 50% of the 329 mothers and fathers had no schooling. Half the fathers were farmers, and another 20% were wage labourers; mothers were housewives. There were no missing data for any of the variables employed, except education of father where n=325, otherwise n=329.

**Table 1.** Means (SD) and *t* values comparing parenting and control families on SES and health (n=329), except education of father (n=325)

Variable	Parenting (n=170)	Control (n=159)	*t* value	p value
Age (months) of child	39.2 (5.3)	39.1 (5.3)	0.25	NS
Education of mother	2.95 (3.6)	3.33 (3.7)	0.96	NS
Education of father	3.26 (3.7)	3.74 (4.5)	1.12	NS
11 assets	5.41 (2.6)	5.01 (2.7)	1.36	NS
Height-for-age	-1.69 (1.1)	-1.76 (1.0)	0.62	NS

NS=Not significant;

SD=Standard deviation;

SES=Socioeconomic status

Although the groups did not differ on the above variables, we correlated these sociodemographic variables with mother and child outcomes. Receptive vocabulary scores correlated negatively with age, indicating that with age children declined in relation to age norms (r=-0.22, p<0.0001). The HOME score correlated positively (p<0.0001), as expected, with education of mother (r=0.32), assets (r=0.29) and education of father (r=0.24). Although height-for-age often correlates with mental development scores, it did not do so in this sample. So, although the socioeconomic variables could not account for outcome differences between the two groups, we covaried the usual variables to provide comparability with other studies: age of child, height-for-age, education of mother, and assets.

### Knowledge of mothers about child development

Knowledge held by mothers about good practices for development was significantly higher in the parenting group, with an effect size d of 0.31 (Table 2). Mothers of boys and girls had similar levels of knowledge. Analyses using both group and a sociodemographic variable cut at the median (assets or education of mother) as predictors found that having attended at least one year of school was related to higher knowledge in both the groups; neither interaction was significant. This meant that both parenting programme and education of mother made independent contributions: unschooled mothers in the parenting programme reached the level of knowledge of schooled mothers in the control group (M's=26.41 and 26.49 respectively).

**Table 2.** Means (SD) and ANCOVA statistics on mother and child (aged 2.5–4.0 years) indicators (n=329)

Indicator (max)	Parenting (n=170)	Control (n=159)	Total (n=329)	Group effect		Sex effect		Interaction	
				F	p value	F	p value	F	p value
Knowledge of mother (39)	27.15 (3.8)	25.83 (4.6)	26.56 (4.2)	9.27	0.0025	2.47	NS	<1	NS
HOME (45)	29.97 (5.2)	28.21 (5.0)	29.11 (5.1)	9.48	0.002	6.16	0.014	<1	NS
Boys	30.5	28.6	29.71						
Girls	29.2	27.9	28.46						
Stimulation (14)	5.78 (3.3)	4.74 (2.8)	5.28 (3.1)	9.45	0.002	11.25	0.0009	<1	NS
Boys	6.04	5.32	5.73						
Girls	5.42	4.26	4.78						
Receptive vocabulary (19)	9.01 (2.6)	9.11 (2.4)	9.06 (2.5)	0.15	NS	<1	NS	4.05	0.04
Boys	9.21	8.77	9.02						
Girls	8.72	9.41	9.10						
Weight-for-height	-1.31 (1.0)	-1.07 (1.1)	-1.19 (1.0)	5.34	0.02	2.22	NS	2.16	NS
Preventive health (5)	4.08 (0.8)	3.84 (0.6)	3.97 (0.7)	9.34	0.002	<1	NS	<1	NS

ANCOVA=Analysis of covariance;

HOME=Home Observation for Measurement of the Environment;

MAX=Maximum score obtainable on the measure;

NS=Not significant;

SD=Standard deviation

### HOME inventory

The total HOME score and the 14-item stimulation subscale were analyzed according to parenting group and sex of child. The parenting mothers obtained significantly higher HOME scores than the control mothers, and the effect size d was 0.34 or small to moderate (Table 2). As a percentage, the mean score of the parenting mothers was 66.7%, although some reached as high as 98%; the mean score of the control mothers was 62.7%. The difference was largely due to the parenting mothers doing better on the stimulation subscale. Although superior, the parenting mothers on average obtained fairly low scores for stimulation, with a mean of 5.78 of 14, or 41.3%, the variation was high. All the mothers provided more stimulation to their sons than to their daughters, according to the main effects for sex on both HOME and stimulation scores; there was no Group × Sex interaction effect. Analyses using both group and a sociodemographic variable cut at the median (assets or education of mother) found significant interaction effects for both assets, *F*(1, 328)=4.00, p=0.046, and education of mother, *F*(1, 328)=5.15, p=0.02 (effect sizes eta^2^ were very small at <0.02 for both). Mothers with assets of 6 or more and mothers who attended school for at least one year benefited more from the programme. Thus, the programme yielded higher HOME scores overall, but particularly among mothers with better resources.

### Mother-child interaction

Verbal interaction of the mothers with their children during the picture task was tallied, and the frequencies for various codes were analyzed according to four levels (Table 3). A 2 (Group) × 2 (Sex) × 4 (Levels of mother's speech) ANCOVA on the number of mother's speech utterances at each level was conducted, with repeated measures on the levels factor. The control mothers talked more with their children but there was no Group × Level interaction (*F*<1). Both used mostly Level 2 speech, questioning, answering, and expanding on detail. Only a few expanded on the child's talk or encouraged the child to talk.

**Table 3.** Means (SD) and ANCOVA statistics on mother-child (aged 2.5–4.0 years) picture dialogue

Level of mother talk	Parenting (n=170)	Control (n=159)	Total (n=329)	Group effect			Level effect		
				*F*	df	p value	*F*	df	p value
Level 0	2.78 (2.6)	3.31 (2.7)	3.04 (2.6)	5.91	327	0.02	11.37	3,960	0.001
Level 1	6.15 (4.1)	7.12 (5.9)	6.61 (5.1)						
Level 2	17.01 (5.6)	17.92 (5.2)	17.45 (5.4)						
Level 3	5.76 (3.6)	6.09 (3.9)	5.92 (3.8)						

Level 0=Evaluate child negatively, off task, disengaged;

Level 1=Command, point to an object, name an object;

Level 2=Question child, answer child, expand on detail beyond naming;

Level 3=Expand on child's behaviour, encourage child to talk, ask to expand, positive evaluation;

ANCOVA=Analysis of covariance;

df=Degrees of freedom;

NS=Not significant;

SD=Standard deviation

### Receptive vocabulary of children

There was no difference between children whose mothers had gone to parenting sessions and controls (Table 2). However, there was a significant Group × Sex interaction (effect size eta^2^=0.013), indicating that boys did better in the parenting group, whereas girls did worse. There is no obvious explanation for this sex difference, except that it matches the amount of HOME stimulation given to boys compared to girls (although HOME and vocabulary correlated r=0.10, p=0.06). Interaction effects of Group × Assets and Group × Education of Mothers were not significant, indicating that the programme had no unique vocabulary benefits for any subgroup of children, except boys.

### Nutritional and physical health status of children

A large proportion of both parenting and control children were moderately or severely stunted (approximately 40%) and wasted (20%). The Group × Sex ANCOVA yielded a group difference in weight-for-height (Table 2). Surprisingly, the parenting group of children had more wasting than the controls, with an effect size d of 0.24 or small. However, weight-for-age showed no group difference (data not shown).

There were significant differences in the sum of five preventive health behaviours (Table 2); in particular, the parenting children were more likely to use a latrine than the control children (30% vs 10.7%). In both groups, the mothers reported that 15% had one of the 10 disabilities, most frequently a delay in acquiring motor milestones which can occur in normal children and may not indicate disability.

### Parenting sessions

On average, the mothers attended 16 (standard deviation 14.3) parenting sessions, with a range of 0 to 40. That is, some mothers signed up but did not attend any session (n=9), and others attended many. Twenty-two percent evaluated the programme as very good, 73% as good, and 5% as more or less good; none stated that it was not good.

Ten parenting sessions were observed during April; so, groups had time to form and stabilize. On average, 17 mothers attended the group session, with a range of 9 to 25, many of whom had babies with them (Table 4). On average, slightly over five specific maternal behaviours were touched on, most of them positive behaviours which the mothers were advised to perform (rather than negative behaviours to avoid), 82% of them were elaborated but only 20% demonstrated, and 5% supported with material props.

**Table 4.** Descriptive features observed during 10 parenting sessions

Variable	Mean (SD)	Range
Who attended		
Mothers present	16.6 (5.5)	9–25
Babies present	10.1 (4.5)	4–17
Specific behaviours	5.20 (1.4)	3–7
% elaborated on	82	
% evaluated as good	84	
% demonstrated	20	
% supported with materials	5	
Problems to enacting advice		
Raised by facilitators	4.00 (1.3)	3–7
Solutions per problem	3.64 (0.96)	1.67–5.0
Solutions offered by facilitators	9.80 (5.1)	2–18
Solutions offered by mothers	4.70 (4.2)	0–14
Raised by mothers	1.10 (1.3)	0–4
Solutions per problem	1.50 (1.5)	0–4
Solutions offered by facilitators	2.67 (1.4)	1–5
Solutions offered by mothers	1.17 (0.4)	1–2
Questions about information/advice		
Raised by facilitators	5.20 (2.3)	1–8
Answers per question	4.17 (1.6)	2–7
Answered by facilitators	8.40 (7.3)	1–20
Answered by mothers	12.60 (8.0)	2–25
Raised by mothers	2.10 (2.0)	0–7
Answered per question	5.37 (4.3)	0–13
Answered by instructors	3.87 (3.9)	1–13
Answered by mothers	0.25 (0.46)	0–1

In some cases where data were skewed, medians might have been more appropriate. However, the medians were often zero, which gave the mistaken impression that no such behaviours occurred. In other cases, only the decimal digit changed by a small amount

Participation was initiated largely by the facilitator who raised problems and posed questions about the topic to help engage the mothers. As seen in Table 4, the facilitator raised 80% of the problems for which both facilitator and mothers offered solutions. When the mothers raised a problem in enacting the behaviour, solutions likewise came mostly from the facilitator and some from the mothers. Problems, then, appeared to be raised by the instructors and were solved by them as part of their lesson plan, although the ranges indicate that in some groups the mothers took more initiative. Questions posed by the facilitator led to a great deal of participation by mothers; the mothers also questioned some information, and the facilitators answered them. So, although the mothers participated, the sessions were largely dominated and directed by the facilitators. Still, overall evaluations given to the session by the observers were very positive: 90% were considered to be participatory, in 100% the facilitator was encouraging, and in 100% the right amount of time was spent giving information (between 40% and 60%).

## DISCUSSION

The first three objectives of the evaluation are discussed first, namely the effects of the parenting intervention on mothers' practices, effects on child health and development outcomes and subgroups that benefited more. The final objective—to assess the method of implementation of parenting activities—is discussed last.

### Mother and child outcomes

The parenting sessions were successful in raising the overall level of knowledge of the mothers about child rearing. Parenting mothers also had higher HOME scores, particularly on the stimulation subscale of the HOME; this was the subscale of items from the learning materials and parent-involvement subscales of the original. Consequently, the parenting programme had positive outcomes on two mother-related measures, namely knowledge about good practices for child development and opportunities for stimulation in the home.

Despite higher scores among parenting mothers, their stimulation scores were low; of the 14 stimulation items, parenting mothers averaged under 6. Items answered affirmatively concerned materials for gross motor and dramatic play and involvement of mothers in structured social games. These rural families lacked many common household assets, and, as such, it was not surprising that they did not have sensory-motor materials or picture books. Still, the emphasis in these programmes is on easily available materials, such as cooking utensils, sticks, and cloth. However, without any models in the village market to copy, the mothers did not know how to combine materials to make them challenging.

Boys received more stimulation than girls in both groups. Boys also had higher receptive vocabulary scores in the parenting group, and girls had lower vocabulary. Until replicated, it would be premature to explain the poorer vocabularies of girls in the parenting group; girls generally excel in verbal skills. The parenting programme addressed gender equality in one session but it may have been insufficient. Bangladesh is considered to be a male-dominated society, where only recently the sex difference in nutritional status and primary education has disappeared (9).

Certain subgroups of mothers benefited more than others from the parenting programme. Unschooled mothers who attended the programme attained the same knowledge score as schooled mothers in the control group. Thus, the parenting sessions compensated for lack of schooling. However, HOME scores in the parenting group were higher only when mothers had resources, either education or household assets. Unschooled mothers and those with few household goods may have been unable to make the leap from information to materials and practices.

The maternal benefits did not reach their children in any way that we observed, except in preventive health. The nutritional status (weight-for-height) of parenting children was worse than that of controls, although weight-for-age was not. The children had similar scores on a measure of receptive vocabulary. This is not to say that there was no variability: some children scored very high (16 of 19) in receptive vocabulary. Educated mothers were more likely to develop these strengths in their children, reinforcing once again, the long-term value of getting girls educated.

Reasons why the knowledge and stimulation benefits did not trickle down to the children require some analysis. Although the mothers showed positively responsive behaviours towards their children during the HOME interview, their communication with their children during the picture task showed little responsiveness to the cognitive and language needs of their children and little verbal elaboration. The prevailing view among parents is that mothers must instruct their children to talk (14). Consequently, most conversation on the picture task consisted of the mother asking “What is this?” and the child answering. If mothers see their role as didactic teachers, they must be more responsive to the abilities of their children to take them to the next level. It is likely that mothers had a low estimation of the language abilities of their children and so talked down to them. Given the proclivities of mothers to use an instructional rather than a scaffolding type of interaction with their children, programmes need to focus more on the know-your-child theme.

### Implementation of parenting programme

Our observations of ongoing parenting sessions identified a number of strengths of the programme. The mothers evaluated the programme positively and could identify ways that the programme had changed them and their practices. A strong infrastructure was in place to recruit and train many village women each year as peer educators to implement the sessions. Extensive training was required to cover all the 40 topics in the parenting manual. The topics were well-chosen, and information in the manual was largely accurate, detailed, and sufficiently colloquial to be understood by most rural, illiterate mothers. The facilitators were observed to be encouraging and friendly in their approach and to allow for participation by the mothers. Giving information accounted for part of the session, and discussing questions and problems accounted for another sizeable part. By raising problems and posing questions, facilitators engaged the mothers actively in the discussion. These qualities contributed to the continued success of the programme.

The most telling clue as to why the children's outcomes did not change came from observation of the sessions themselves. Although five specific practices were mentioned, only one of the five practices was demonstrated and very rarely with material props. Babies were always present at these sessions and could have been used for demonstrating a point, such as where signs of health or disease show up and how to encourage play and conversation. Other more successful programmes, such as the Turkish Early Enrichment Project in Istanbul (4) had mothers rehearse the learning games they would play with their children during the coming week. This built on the mothers' desire to ‘teach’ their children. Although the Turkish children were older and the mothers were more literate, what may be critical is the focus on specific cognitive and language-learning games provided in worksheets, the agreement among mothers to practise the games and skill-building through role-playing.

In addition to rehearsing the new practices, effective behaviour-change programmes provide problem-solving skills and peer support (18). As was learned in the past with nutrition-education programmes, mothers run into barriers when implementing the advice; yet, they are the ones to generate solutions that make sense to them (28). Friends and peer educators are most useful not simply for information support about alternative solutions but also for emotional support that builds confidence in one's problem-solving efforts.

The limitations of the cross-sectional design of the present study prevent conclusions about how the mothers and children changed from start to finish of the programme. We looked only at the endpoint in comparison with non-participating villages. Although the neighbouring control villages were closely matched to the intervention villages in sociodemographic variables, their baseline similarities could not be assessed. It is possible that the intervention villages were initially worse or better-off as a result of the involvement of organization in community development. Also, the use of tests developed and used in Western countries may have handicapped the mothers and their children. The validity of the HOME, modified to suit the kinds of stimulation available and promoted by the parenting programme, was confirmed through correlations with expected parental education and family resources. Measures of receptive vocabulary are frequently used in developing countries with modification (29) and were particularly relevant here with the focus on language stimulation by the mothers. Mother-child interaction tasks are commonly used for direct observation of verbal interaction and tap into items on the HOME. The picture-talk task showed wide variation, indicating that some mothers used higher-level talk. Still, it is open to criticism.

In conclusion, the mothers in the parenting programme achieved higher levels of knowledge than the control mothers and provided more stimulation for their children. However, the children did not show benefits in nutritional status or language development. This may be due to limitations in the curriculum, which focused more on increasing knowledge of mothers than on improving their practices. Strategies for behaviour change need to be part of the curriculum, such as role plays and rehearsing the practice with one's child, and peer support in solving each mother's specific problems in implementing advice. Much needs to be learned about the effectiveness of parenting programmes around the world to develop a model that benefits children and mothers. Organizations offering such programmes are encouraged to allocate a percent of their budget to evaluation research to contribute to this effort.

## ACKNOWLEDGEMENTS

The research was conducted while the author was at the International Centre for Diarrhoeal Disease Research, Bangladesh (ICDDR,B). The author acknowledges the financial support of Plan International Bangladesh which funded this independent research (Grant No. 2003-029). The grant was administered by ICDDR,B, where the author spent two years on sabbatical leave. The author was ably assisted in the training and conduct of the study by Sadika Akhter. Data were carefully and competently collected by the following research assistants: Faizun Nessa, Farah Deeba, Abida Sultana, Mousumi Biswas, Zaheda Parvin, Md. Noor Hasan, Ashraf Khan Eusufzi, Shamsunnahar Chonda, and Nasir Uddin. Data entry was managed by Farhana Yasmin and instruments translated by Farhana Tofail. Finally, the author is grateful to the participants, including children, mothers, community facilitators, and supervisors. ICDDR,B acknowledges with gratitude the commitment of Plan International Bangladesh to the Centre's research efforts.
